# Doxorubicin-loaded platelets conjugated with anti-CD22 mAbs: a novel targeted delivery system for lymphoma treatment with cardiopulmonary avoidance

**DOI:** 10.18632/oncotarget.16871

**Published:** 2017-04-06

**Authors:** Peipei Xu, Huaqin Zuo, Rongfu Zhou, Fan Wang, Xu Liu, Jian Ouyang, Bing Chen

**Affiliations:** ^1^ Department of Hematology, Drum Tower Hospital, School of Medicine, Nanjing University, Nanjing, Jiangsu, 210093, P. R. China

**Keywords:** platelet, anti-CD22 mAbs, doxorubicin, drug delivery system, lymphoma

## Abstract

B-cell lymphoma accounts for approximately 85% of all adult non-Hodgkin's lymphoma cases. Doxorubicin (DOX) is an indispensable drug for the treatment of non-Hodgkin's lymphoma. However, DOX causes severe cardiotoxicity, which limits its use in conventional treatment strategies. In this study, we developed a novel drug delivery system for lymphoma treatment: DOX-loaded platelets that were conjugated with anti-CD22 monoclonal antibodies (mAbs) (DOX–platelet–CD22). Platelets are bio- and immune-compatible drug carriers that can prolong the circulation time of drugs. Anti-CD22 mAb-labeled platelets can precisely deliver DOX to tumor cells. Our *in vitro* and *in vivo* experiments showed the enhanced antitumor activity and attenuated cardiotoxicity of DOX when delivered as DOX–platelet–CD22. Compared with other delivery systems, the uptake of DOX–platelet–CD22 by macrophage-like cells decreased. Moreover, DOX–platelet–CD22 showed platelet properties, such as tumor cell-induced platelet aggregation. Therefore, targeted chemotherapy that is mediated by DOX–platelet–CD22 is a promising option for lymphoma treatment.

## INTRODUCTION

B-cell lymphoma, a group of heterogeneous tumors that is associated with malignant B-cell monoclonal amplification, accounts for approximately 85% of adult non-Hodgkin's lymphoma (NHL) cases [1, 2]. In turn, NHL accounts for approximately 90% of all lymphomas. NHL incidence has dramatically increased in recent years [3]. B-cell lymphoma, a highly heterogeneous hematologic malignancy, manifests different clinical and biological characteristics. Despite the progress in treatment response, the five-year survival rate of high grade B-cell NHL is less than 50% [4]. Chemotherapy remains the first-line treatment for B-cell lymphoma. However, cytotoxic drugs cause various adverse reactions in normal tissues and cells, thus limiting the applied dosage of chemotherapeutic agents and hampering their effects on tumor cells. For example, doxorubicin (DOX), an important antitumor agent in lymphoma, causes severe cardiotoxicity [5].

Therefore, crucial requirements for effective cancer treatment include tumor-specific drug accumulation and the protection of normal tissues from cytotoxic effects. Various drug delivery systems, including liposomes, nanocarriers, and biological drug carriers, have been developed in recent years [6–10]. Although liposomes once received extensive research attention, their poor storage stability and complicated synthesis procedures have impeded their clinical applications [11]. In addition, the wide application of nanocarriers is restricted by their nanotoxicity and limited biodegradability [12].

Biological drug carriers, such as erythrocytes, macrophages, and platelets, have attracted great interest because of their superior biocompatibility, prolonged circulation time, and simple production process [13–15]. In this work, native platelets were used as drug vehicles for DOX delivery in lymphoma treatment. Platelets have several inherent properties that make them favorable drug delivery systems: first, platelets are abundant and do not require complicated production procedures [16]. Native platelets, however, are rarely directly applied as a drug carrier. Second, platelets possess a 7–10 day lifespan and can uptake nearby small molecules to shelter them from immune surveillance [17], thereby prolonging their circulation time. Third, platelets can be activated by tumor cells via tumor cell-induced platelet aggregation [18], which causes platelets to release their drug load around the tumor site. Moreover, the large gaps between endothelial cells in the tumor vasculature cause leaky blood vessels (termed the “enhanced permeability and retention effect”); this effect enhances the suitability of platelets as a drug delivery vehicle for tumor treatment [19, 20]. Accordingly, platelets deliver the loaded drugs to tumor cells, thus reducing negative reactions in normal tissues.

The challenging specific and precise targeting of platelets limits their efficacy as drug carriers. Monoclonal antibodies (mAbs), by contrast, are highly specific and precise targeting systems. Conjugating drugs to tumor-specific antibodies can direct drugs to tumor cells, demonstrating their potential use for targeted drug delivery [21]. CD22 has attracted particular attention as a chemoimmunoconjugate for targeted cytotoxic drug delivery to tumor cells because antibodies that are bound to CD22 can be rapidly internalized [22, 23].

**Figure 1:**
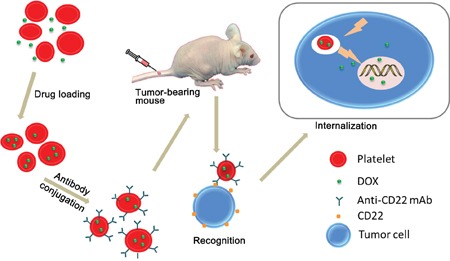
Schematic of DOX-platelet-CD22 preparation and mechanism of its enhanced anti-tumor activity DOX-platelet-CD22 can specifically target tumor cells through antigen-antibody binding and is then internalized to exert cytotoxic effects.

In this work, we propose using DOX-loaded platelets that were conjugated with anti-CD22 antibodies (DOX–platelet–CD22) as a novel drug delivery system for the treatment of B-cell NHL. Figure 1 shows the schematic of the putative mechanism that underlies this drug delivery process. This novel system possesses several ideal qualities that minimize adverse effects and maximize therapeutic potential, including excellent biocompatibility, long circulation time, and precise targeting. Characterization revealed that the novel drug delivery system maintained the natural properties of platelets. We assessed the *in vitro* and *in vivo* antitumor efficacy of the system. Our results consistently showed that the therapeutic effects of DOX loaded on DOX–platelet–CD22 significantly improved, whereas toxicity to normal tissues was reduced. Overall, the novel DOX–platelet–CD22 drug delivery system offers a promising new therapeutic option for lymphoma.

## RESULTS

### Characterization of DOX–platelet–CD22

**Figure 2:**
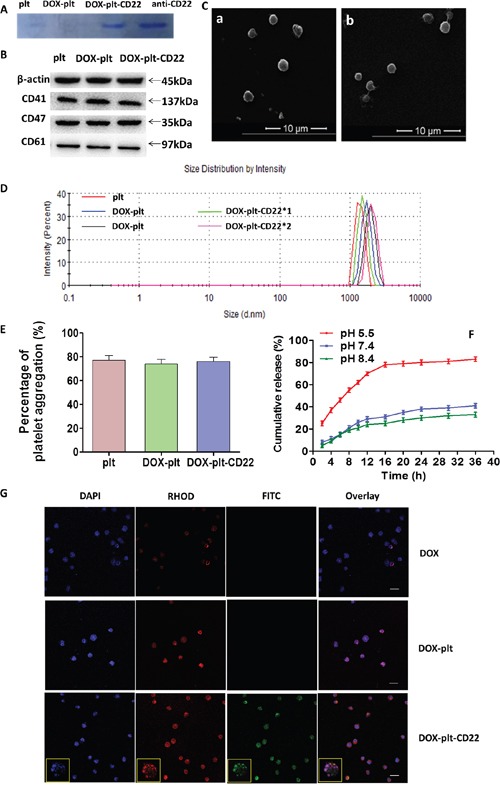
Characterization of DOX-platelet-CD22 **(A)** Coomassie Brilliant Blue staining after protein electrophoresis of native platelets, DOX-platelet, DOX-platelet-CD22 and anti-CD22 mAbs. **(B)** Representative western blot protein bands from the three platelet groups. **(C)** a, SEM image of native platelets; b, SEM image of DOX-platelet-CD22. **(D)** The sizes of native platelets, DOX-platelet and DOX-platelet-CD22 determined by DLS. **(E)** ADP-induced aggregation percentage of washed native platelets, DOX-platelet and DOX-platelet-CD22 at 5 min. **(F)** Cumulative *in vitro* DOX release behaviors at pH 5.5, 7.4 and 8.4. **(G)** Confocal microscopy images of Raji cells treated with DOX, DOX-platelet and DOX-platelet-CD22 (scale bar: 10 μm, ×200; insert: ×400) after DAPI staining. Anti-CD22 mAbs were cross-linked with FITC, and DOX autofluorescence is red. **Abbreviations**: DOX, doxorubicin; plt, platelet; SEM, scanning electron microscope; ADP, adenosine diphosphate; DLS, dynamic light scattering; FITC, fluorescein isothiocyanate.

Different concentrations of DOX were used to achieve the optimal drug loading (DL) and encapsulation efficiency (EE), which were measured by high-performance liquid chromatography. The maximum DL and EE were 46.3% and 86.6%, respectively, at a DOX concentration of 0.1 mmol/L. This concentration was used for subsequent experiments. Protein bands of native platelets, DOX–platelet, DOX–platelet–CD22, and anti-CD22 mAbs were stained with Coomassie Brilliant Blue, the results (Figure 2A) indicated that anti-CD22 mAbs were successfully conjugated to DOX–platelet. The conjugation of anti-CD22 mAbs to DOX–platelet was verified by the green fluorescence on the surface of Raji cells treated with DOX–platelet–CD22, which were generated by cross-linking anti-CD22 mAbs with fluorescein isothiocyanate (FITC) (Figure 2G).

The levels of the platelet membrane proteins CD41, CD47, and CD61 were measured by Western blot. As shown in Figure 2B, the native platelets, DOX–platelet, and DOX–platelet–CD22 did not have significantly different levels of platelet membrane proteins. The influence of DOX and anti-CD22 mAbs on platelet morphology was assessed by scanning electron microscopy (SEM). As Figure 2C shows, no significant changes were observed between native platelets and DOX–platelet–CD22. Furthermore, the sizes of DOX–platelet and DOX–platelet–CD22 were similar to those of native platelets (Figure 2D). The platelet aggregation assay revealed (Figure 2E) that there were no significant differences in aggregation function among DOX–platelet–CD22, DOX–platelets, and native platelets. The cumulative release of DOX from DOX–platelet–CD22 is shown in Figure 2F. DOX was released most rapidly at pH 5.5, an acidic condition, with approximately 83% of the drug released within 36 h. At pH 7.4 and 8.4, however, DOX was released at considerably slower rates than that at pH 5.5. This finding suggests a pH-triggered release behavior.

### Cellular uptake and cytotoxicity of DOX–platelet–CD22 *in vitro*

**Figure 3:**
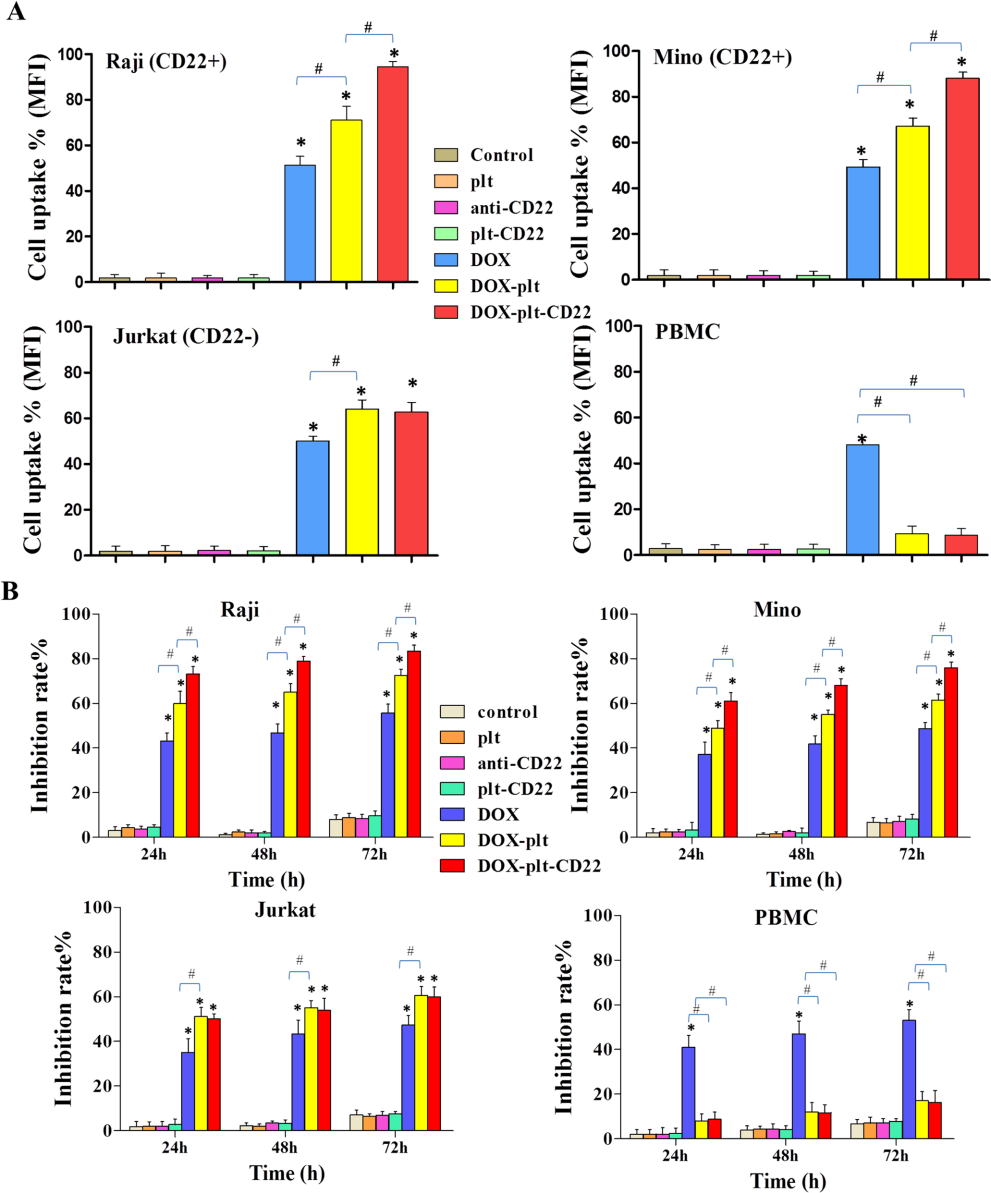
(A) DOX uptake by different cells FCM was used to determine the uptake of DOX by Raji, Mino, Jurkat and PBMC cells. The bar graph shows comparative intracellular DOX uptake in different cells for each treatment group, as quantified by MFI. (*P<0.05 when compared with controls, #*P*<0.05). **(B)** Growth inhibition in cells with different treatments for 24, 48 and 72 h. (* *P*<0.05 when compared with controls at 24, 48 and 72 h; #*P*<0.05) **Abbreviations**: plt, platelet; DOX, doxorubicin; FCM, flow cytometry; MFI, mean fluorescence intensity; PBMC, peripheral blood mononuclear cell.

To determine if the platelets that were conjugated with anti-CD22 mAbs can exclusively facilitate DOX accumulation in CD22+ tumor cells, drug concentration in different cells was determined by flow cytometry (FCM) analysis of intracellular fluorescence intensity. As shown in Figure 3A, intracellular DOX concentration significantly increased in CD22+ tumor cells (Raji and Mino cells) that were treated with DOX–platelet–CD22 compared with those in cells treated with DOX alone or DOX–platelet. Meanwhile, no significant difference in intracellular DOX concentration was observed between CD22-tumor cells (Jurkat cells) that were treated with DOX–platelet–CD22 and DOX–platelet. Furthermore, the growth inhibition rate of normal cells (PBMC) treated with DOX-platelet or DOX–platelet–CD22 was significantly lower than that treated with free DOX (*P*<0.05). These findings are consistent with the results of intracellular DOX concentration.

In order to determine cells viability after treatment, different cells were treated with anti-CD22, platelet–CD22, DOX, DOX–platelet or DOX–platelet–CD22. At 24, 48, and 72 h after treatment, the cell viability were examined using c*ell counting kit-8* (CCK-8) assay. The results demonstrated that the viability of CD22+ tumor cells (Raji and Mino cells) that were treated with DOX–platelet–CD22 significantly decreased compared with those treated with DOX alone or DOX–platelet, as illustrated in Figure 3B (*P*<0.05). Moreover, no significant difference in cell viability was observed between cells that were treated with anti-CD22 mAbs and the control. This finding indicates that anti-CD22 mAbs helps achieve targeted delivery without interfering with therapeutic effects. DOX–platelet–CD22-treated Jurkat cells (CD22-tumor cells) exhibited no significant difference in cell viability compared with those treated with DOX–platelet. Additionally, the growth inhibition rates of PBMC (normal cells) that were treated with DOX–platelet or DOX–platelet–CD22 was significantly lower than that of cells treated with free DOX (*P*<0.05). These findings are consistent with the results of intracellular DOX concentration.

### Increases in apoptosis and cell cycle arrest by DOX-platelet-CD22

**Figure 4:**
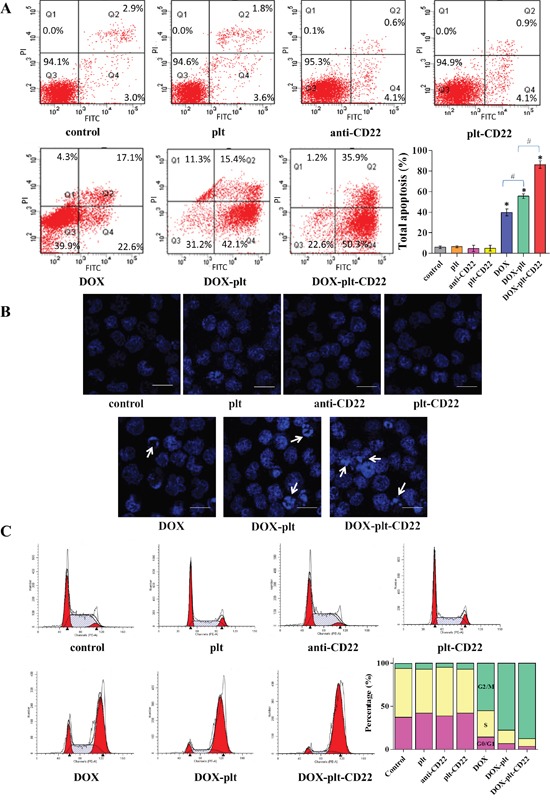
Apoptosis and cell cycle state of Raji cells **(A)** FCM was used to investigate the apoptosis of Raji cells following the different treatments; the bar graph shows the comparative apoptosis levels of Raji cells from different treatment groups. (*P<0.05 when compared with controls, #*P*<0.05). **(B)** Fluorescence microscopy images of Raji cells after DAPI staining (scale bar: 10 μm; ×400). Cell apoptosis is indicated by “→”. **(C)** Cell cycle state of Raji cells analyzed by FCM. The bar graph shows the percentage of cells in G0/G1, S, and G2/M phases under different treatment conditions. **Abbreviations**: FCM, flow cytometry; DOX, doxorubicin; plt, platelet.

To determine whether the apoptosis of Raji cells is increased by DOX-platelet-CD22, FCM was utilized to quantify apoptosis of Raji cells after different treatments (Figure 4A). The total apoptosis percentages were 5.9%, 6.4%, 4.7%, 5.0%, 39.7%, 55.5%, and 86.2% in untreated cells, cells treated with native platelets, anti-CD22 mAbs, platelet-CD22, DOX, DOX–platelet, and DOX–platelet–CD22, respectively. Apoptosis levels among cells that were treated with DOX–platelet–CD22, DOX–platelet, and DOX alone were significantly different (*P*<0.05).

The morphological changes in Raji cell nuclei after different treatments were observed by fluorescence microscopy. Chromatin stained homogenously in control cells and those treated with native platelets, as shown in Figure 4B. Meanwhile, an apoptotic appearance, which was characterized by chromatin condensation and nuclear fragmentation, was observed in cells that were treated with DOX alone, DOX–platelet, and DOX–platelet–CD22. This apoptotic appearance was particularly pronounced in the latter, thus demonstrating that DOX–platelet–CD22 improved the ability of DOX to induce cell apoptosis.

FCM was utilized to analyze the cell cycle stage of Raji cells that were treated with anti-CD22, platelet–CD22, DOX alone, DOX–platelet, or DOX–platelet–CD22. As shown in Figure 4C, the proportion of tumor cells in the G2/M phase significantly increased in cells treated with DOX–platelet–CD22 compared with that in cells treated with DOX–platelet. Additionally, the proportion of tumor cells in the G2/M phase significantly increased upon treatment with DOX–platelet compared with those in cells treated with DOX alone. These findings indicate that anti-CD22 mAbs and platelets can enhance the therapeutic effects of DOX.

### Mechanisms for DOX-platelet-CD22-induced apoptosis

**Figure 5:**
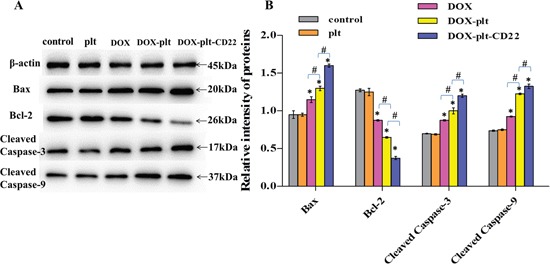
Protein expression of apoptosis-related genes, as quantified by western blotting (*P<0.05 when compared with controls, #*P*<0.05) **Abbreviations**: DOX, doxorubicin; plt, platelet.

To further understand the mechanism that underlies apoptosis in Raji cells, western blot was performed to analyze the expression levels of Bcl-2-associated X protein (Bax), B-cell lymphoma-2 (Bcl-2), cleaved caspase-3, and cleaved caspase-9 in Raji cells treated with different agents. Compared with the controls, the protein expression levels of Bax, cleaved caspase-3, and cleaved caspase-9 were upregulated in cells that were treated with DOX, DOX–platelet, and DOX–platelet–CD22, as shown in Figure 5. This upregulation was particularly pronounced in cells that were treated with DOX–platelet–CD22 (*P*<0.05). Bcl-2 expression was downregulated in cells that were treated with DOX alone compared with control cells. Bcl-2 expression was further downregulated when DOX was carried by platelets that were conjugated with anti-CD22 mAbs. These results indicate that platelets and mAbs can improve the antitumor activity of DOX.

### Anti-tumor activity of DOX-platelet-CD22 *in vivo*

**Figure 6:**
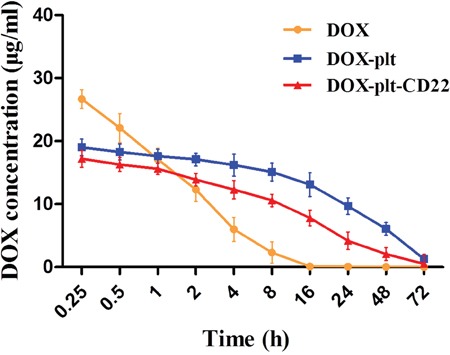
Plasma concentration of DOX *in vivo* Plasma concentrations of DOX over time in tumor-bearing mice after injection with free DOX, DOX-platelet or DOX-platelet-CD22. **Abbreviations**: DOX, doxorubicin; plt, platelet.

Plasma DOX concentration over time of mice were determined by spectrofluorometry after they were intravenously administered with free DOX, DOX–platelet or DOX–platelet–CD22. The results are shown in Figure 6. Free DOX was rapidly cleared from blood circulation with a short half-life (t1/2 = 1.87±0.41 h). Meanwhile, DOX in the forms of DOX–platelet and DOX–platelet–CD22 showed a prolonged blood circulation time with a long half-life (t1/2 = 24.11±1.02 h, t1/2 = 19.23±1.25 h).

**Figure 7:**
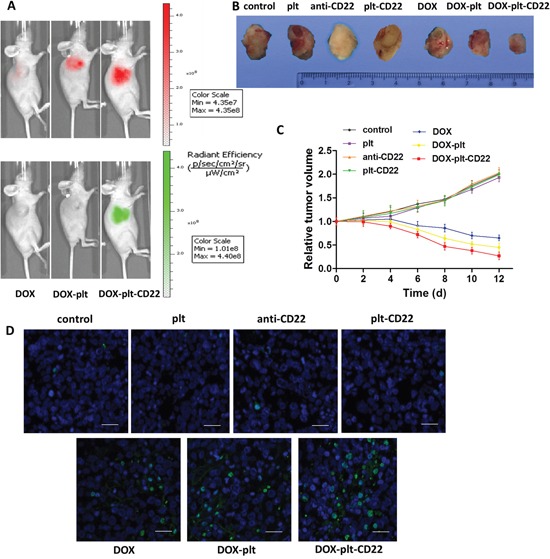
Effects of DOX-platelet-CD22 *in vivo* **(A)** Fluorescence images of tumor-bearing mice after different treatments. The upper is the DOX fluorescence and the lower is the fluorescence of FITC-labelled anti-CD22 mAbs. **(B)** The final tumor volume of mice under different treatment conditions. **(C)** The changes in relative tumor volume over a period of 12 days after the administration of different treatments. **(D)** TUNEL staining of tumor tissue sections after different treatments (scale bar: 20 μm). DAPI was used to stain nuclei with fluorescein for TUNEL. **Abbreviations**: DOX, doxorubicin; plt, platelet; TUNEL, TdT-mediated dUTP nick end labeling.

Next the *in vivo* distribution of DOX–platelet–CD22 in tumor-bearing mice was characterized using optical imaging. DOX fluorescence intensity in tumor sites increased sequentially from left to right, as shown in Figure 7A. The DOX–platelet–CD22 group exhibited the most intense fluorescence. The results revealed that loading DOX on platelets conjugated with anti-CD22 mAbs greatly improved DOX accumulation in tumor tissues. Moreover, DOX–platelet–CD22 showed excellent targeting effect compared with DOX–platelet and DOX alone.

To investigate the therapeutic effects of DOX–platelet–CD22 *in vivo*, mouse tumor size was measured every two days after different treatments. The average tumor volume of mice that were treated with saline or native platelets increased continuously, as shown in Figure 7C. By contrast, the average tumor size of mice that were treated with DOX alone, DOX–platelet, or DOX–platelet–CD22 steadily decreased. The final volumes of tumors from the DOX–platelet–CD22 group were also significantly smaller than those of tumors from the DOX–platelet group (Figure 7B, Supplementary Figure 1). The results of TdT-mediated dUTP nick end labeling (TUNEL) staining for tumor tissues (Figure 7D) also showed that apoptosis in tumor tissues from mice that were treated with DOX–platelet–CD22 obviously increased.

### Reduced toxicity of DOX-platelet-CD22 towards normal tissues

**Figure 8:**
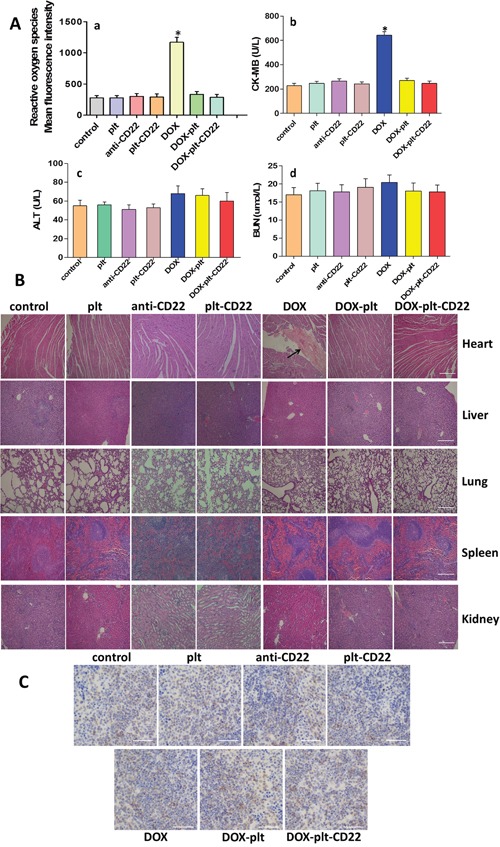
Toxicity in normal tissues obtained from animals in different treatment groups **(A)** a, ROS in myocardial cells was analyzed by FCM; b-d, alanine transaminase (ALT), blood urea nitrogen (BUN), and creatine kinase-MB (CK-MB) were monitored on day 12 after treatment. (*P<0.05 when compared with control). **(B)** Hematoxylin-eosin staining of heart, liver, lung, spleen and kidney tissues obtained from animals in the different treatment groups (scale bar: 200μm; ×40). The histopathological changes are indicated by “→”. **(C)** Immunohistochemical staining for B cells in spleens from different groups (scale bar: 100μm; ×100). **Abbreviations**: DOX, doxorubicin; plt, platelet; ROS, reactive oxygen species; FCM, flow cytometry.

Reactive oxygen species (ROS) induce cell damage. ROS production in myocardial cells from treated mice was measured by FCM (Figure 8Aa). The results showed that myocardial cells from DOX-treated mice exhibited a considerably higher ROS level than others, which demonstrates that conjugating platelets with anti-CD22 mAbs can decrease DOX cardiotoxicity.

The serum concentrations of creatine kinase-MB (CK-MB), alanine transaminase (ALT), and blood urea nitrogen (BUN) of tumor-bearing mice were measured on day 12 after treatment. Figures 8Ab–8Ad show that only the CK-MB concentration in the DOX group was significantly different from that in the control group. Major organs were isolated from treated mice and histopathological analysis was performed on all tissues to assess the histopathological changes (Figure 8B). No abnormal histopathological changes were evident in any organs in the control, platelet, DOX–platelet, and DOX–platelet–CD22 groups. However, myocardial injury was observed in the DOX group. These results suggest that loading drugs on platelets and anti-CD22 mAbs can protect the heart from exposure to cytotoxic drugs. Furthermore, immunohistochemical staining was performed on the paraffin-embedded sections of spleens from each group with anti-CD19 mAbs to quantify B cells. Figure 8C shows that anti-CD22 mAbs and platelets that were conjugated with anti-CD22 mAbs did not significantly affect normal B cells and will not reduce normal B cells.

### Apoptosis mechanisms of DOX–platelet–CD22 antitumor effects *in vivo*

**Figure 9:**
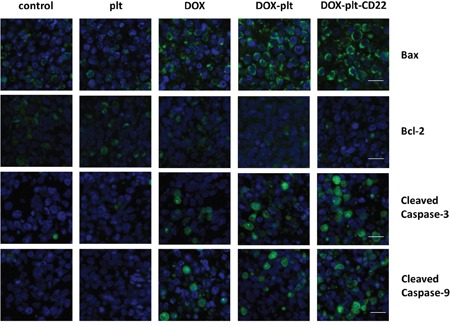
Immunofluorescence staining of tumor sections (scale bar: 10μm; ×400) The nuclei were stained with DAPI; FITC-conjugated antibodies are used as the secondary antibodies for the primary antibodies for Bax, Bcl-2, cleaved caspase-3 and cleaved caspase-9. **Abbreviations**: DOX, doxorubicin; plt, platelet.

Tumors were harvested from mice after treatment and were subjected to immunofluorescence assay to obtain better insight into the mechanism by which DOX–platelet–CD22 inhibited tumors. Bax, cleaved caspase-3, and cleaved caspase-9 exhibited a strong green fluorescent staining pattern in cells that were treated with DOX alone, DOX–platelet, and DOX–platelet–CD22, as shown in Figure 9. These staining intensities increased sequentially from left to right. By contrast, the fluorescence intensity of Bcl-2 decreased sequentially from control cells to those treated with native platelets, DOX alone, DOX–platelet, and DOX–platelet–CD22. This pattern is consistent with the Western blot data that were obtained from Raji cells *in vitro*. These results indicate that the genes that encode Bax, Bcl-2, caspase-3, and caspase-9 are related to the anticancer activity of DOX.

## DISCUSSION

Chemotherapy remains the mainstay of B-cell lymphoma treatment [24]. However, chemotherapy is a double-edged sword that attacks the tumor aggressively as possible while causing severe, dose-dependent side effects. Moreover, cytotoxic drugs are nonspecific to tumor cells, thus influencing not only malignant cells but also normal tissues [25]. Consequently, anticancer drugs are often administered at suboptimal doses, which may not meet therapeutic expectations. Therefore, drug delivery vehicles are crucial in alleviating adverse reactions and improving therapeutic effects.

Platelets are promising drug carriers given their inherent biological properties. Several studies have reported the use of platelets: by taking advantage of their hemostatic function, platelets have been used to directly deliver coagulation factors to sites of vascular injury [26]. Moreover, DOX-loaded platelets exhibit greater antitumor activity than free DOX in Ehrlich ascites carcinoma [17]. Our previous work also showed that platelets improve the therapeutic effects of DOX on lymphoma. All these studies indicated that native platelets are favorable drug carriers. However, more studies on platelets as drug carriers are needed. In addition to the above studies, platelet membranes can be utilized to cloak nanoparticles for drug delivery; however, the procedure is complex and is associated with unavoidable nanotoxicity [27]. Accordingly, the use of native platelets as drug vehicles continues to warrant our efforts and investigation. Drug delivery by platelets is nonspecific and imprecise. Thus, we proposed to exploit tumor-specific antibodies for precise targeting. To the best of our knowledge, we are the first to propose mAbs-conjugated native platelets as a targeted drug delivery system. In this study, anti-CD22 mAbs were utilized for targeted drug delivery. As a transmembrane glycoprotein, CD22 is selectively expressed on the surface of B cells and their malignant counterparts [28]. Once antibodies bind to CD22, the antibody–CD22 complex is rapidly internalized, making CD22 ideal for targeted drug delivery [29]. Raji cells are derived from Burkitt lymphoma, a form of NHL that is composed of B lymphocytes. In Burkitt lymphoma, CD22 is highly expressed on the cell surface. Herein, we used mAbs against CD22 as a delivery vehicle to precisely direct DOX-loaded platelets to tumor sites for the treatment of lymphoma.

The results of platelet characterization, including SEM images of platelet morphology, Western blot of platelet membrane proteins, and the analysis of platelet aggregation, indicate that the mAbs-coupled drug-loaded platelets retain the properties of native platelets. These drug-loaded platelets lack obvious morphological and functional changes, thus demonstrating that the drug-loaded platelets are stable and favorable candidates for drug delivery systems.

The *in vitro* anti-tumor effects of DOX–platelet–CD22 were subsequently evaluated. The increased intracellular DOX concentration indicated that delivery with platelets and anti-CD22 mAbs increased DOX uptake by Raji cells. Moreover, using platelets congjugated with anti-CD22 mAbs as drug delivery vehicles increased the cytotoxic effects of DOX against Raji cells. The results of both growth inhibition and Raji cell apoptosis under different treatment conditions revealed that the cytotoxic effects of DOX–platelet and DOX–platelet–CD22 were superior to that of free DOX. This enhanced cytotoxicity was particularly pronounced in cells that were treated with DOX–platelet–CD22. In addition, the cell cycle test revealed that the abundance of Raji cells in the G2/M phase clearly increased following treatment with DOX–platelet–CD22. Altogether, our data indicate that anti-CD22 mAbs and platelets facilitate DOX accumulation in tumor cells, which inhibits tumor growth by blocking cell cycle progression, suppressing proliferation, and inducing apoptosis. These actions improve the therapeutic effects of DOX without altering its concentration.

A tumor-bearing mouse model was established to estimate the *in vivo* effects of DOX–platelet–CD22. Optical imaging is widely used to track and analyze drug distribution *in vivo* [30]. In this study, we utilized the auto-fluorescence of DOX and FITC-labeled anti-CD22 mAbs for *in vivo* imaging. DOX accumulation at tumor sites in the DOX–platelet–CD22 group was the highest. This result suggests that DOX–platelet–CD22 can target tumor tissues. Thus, therapeutic effects are enhanced and side effects on normal tissues are reduced. The tumor volumes of DOX, DOX–platelet, and DOX–platelet–CD22 mice significantly decreased compared with those of the controls. Moreover, tumor size was the smallest in the DOX–platelet–CD22 group. This finding illustrates that DOX–platelet–CD22 remarkably improves the efficacy of DOX in lymphoma treatment. Therefore, DOX–platelet–CD22 targets tumor sites and decreases the dosage of cytotoxic drugs necessary for chemotherapeutic activity. This phenomenon results in less drug accumulation in normal tissues, thus alleviating the side effects caused by anti-tumor drugs. Dose-dependent cardiotoxicity, the most severe side effect of DOX, critically limits its use. ROS production is related to oxidative stress injury. Serum concentrations of CK-MB, ALT, and BUN also reflect myocardial damage, liver function, and renal function, respectively. Our results show that myocardial damage occurred only upon treatment with free DOX. This finding demonstrates the ability of platelets and anti-CD22 mAbs to attenuate the adverse effects of DOX without causing morphological or functional changes in normal tissues and organs.

To investigate the possible mechanism of action of DOX–platelet–CD22, the expression levels of the apoptosis-related proteins Bax, Bcl-2, cleaved caspase-3 and cleaved caspase-9 were detected by Western blotting and immunofluorescence staining. Caspases (cysteine-aspartic proteases), including caspase-3 and caspase-9, are a family of proteins that induce programmed cell death [31]. The Bcl-2 family activates cell apoptosis by either pro-apoptotic or anti-apoptotic activities. Bax is a pro-apoptotic regulator that antagonizes the anti-apoptotic effect of Bcl-2 [32]. Bax improves the permeabilization of the mitochondrial outer membrane, subsequently activating downstream caspase-9 [33]. The final stage of apoptosis occurs once caspase-9 initiates the cleavage of procaspase-3 [34]. Compared with the controls, the protein expression levels of Bax, cleaved caspase-3, and cleaved caspase-9 were upregulated in cells that were treated with free DOX and DOX–platelet. This upregulation was significantly pronounced in cells that were treated with DOX–platelet–CD22 (*P*<0.05). By contrast, Bcl-2 expression was downregulated when DOX was delivered by platelets that were conjugated with anti-CD22 mAbs. The variation in expression, as revealed by immunofluorescence staining, was in accordance with the findings of our Western blot assay of Raji cell proteins. These results indicate that Bax, Bcl-2, caspase-3, and caspase-9 are related to the anti-cancer activity of DOX–platelet–CD22. Platelets and anti-CD22 mAbs can increase DOX accumulation in tumor cells. Thus, the cytotoxic effects of DOX on tumors are enhanced by promoting tumor cell apoptosis through a Bcl-2/caspase-mediated apoptosis signaling pathway.

In summary, our proposed DOX–platelet–CD22 drug delivery system is biocompatible with normal tissues and specific to tumor cells. This system enhances the therapeutic effects and reduces the adverse effects of DOX, thus representing a novel therapy for lymphoma.

## MATERIALS AND METHODS

### Materials

All reagents used in this study were analytically pure and required no further purification. DOX was obtained from Kanion Pharmaceutical Co. Ltd. (Jiangsu, China). Fetal bovine serum (FBS) was purchased from Wisent Inc. (Montreal, Canada). Roswell Park Memorial Institute 1640 medium was purchased from Thermo Fisher Scientific (MA, USA). The CCK-8 assay, annexin V-FITC apoptosis detection kit, and 4′,6-diamidino-2-phenylindole (DAPI) staining solution were purchased from Beyotime Institute of Biotechnology (Jiangsu, China). The Cycletest™ Plus DNA Reagent Kit and mAbs for CD22, CD41, CD47, CD61, Bax, Bcl-2, cleaved caspase-3, cleaved caspase-9, and β-actin were obtained from CST (MA, USA). H2DCFDA was bought from KeyGEN (Jiangsu, China).

All animal studies were in compliance with the Guidelines of the Animal Ethics Committee of the Affiliated Drum Tower Hospital of Nanjing University Medical School (Nanjing, China). BALB/c-nude mice (four to six weeks, 18–22 g) were purchased from Shanghai National Center for Laboratory Animals (Shanghai, China). Animals were maintained in specific pathogen-free facilities with food and water supplied ad libitum.

### Platelet isolation

All volunteers were offered written informed consent. With the permission of the institutional ethics committee, fresh whole blood was drawn from the veins of healthy volunteers. The whole blood was anti-coagulated with sodium citrate and centrifuged at 200 ×*g* for 10 min. The resultant supernatant was centrifuged at 1800×*g* for 20 min. The resulting precipitate was washed with PBS to obtain purified platelets with more than 96% purity (Supplementary Figure 2).

### Preparation of DOX–platelet–CD22

Different DOX concentrations were mixed with platelets (0.2 × 10^9^/mL) in a range of volume ratios. The mixture was incubated under constant shaking at 100 rpm at 37°C for 1 h away from light. Afterwards, DOX-loaded platelets (DOX–platelet) were obtained using a Sepharose 2B column. The highest degree of DL and EE was determined by fluorescence microscopy and high-performance liquid chromatography.

Ten microliters of methyl methacrylate-butadiene-styrene (MBS) was added to 1 mL of DOX–platelet. The mixture was incubated under constant shaking of 600 rpm at 20°C for 1 h. After incubation, the mixture was purified by centrifugation at 12,000 ×*g* for 10 min. Then, the precipitate was dispersed in 500 μL PBS. Anti-CD22 mAbs at a concentration of 1 mg/mL. Antibodies were thiolated with a 50-fold molar excess of Traut's reagent for of 2 h under constant shaking of 600 rpm at 20°C. The thiolated antibodies were purified using a D-Salt Dextran Desalting column and subsequently dispersed in 500 μL PBS. For the conjugation reaction, 500 μL of the described cross-linker-activated DOX–platelet suspension was added to 500 μL of the purified thiolated anti-CD22 mAbs. The mixture was incubated for 12 h and re-purified via centrifugation and re-dispersion thrice. Ultimately, DOX–platelet–CD22 was obtained.

### Characterization of DOX–platelet–CD22

After protein electrophoresis, protein bands of native platelets, DOX–platelet, DOX–platelet–CD22, and anti-CD22 mAbs were stained with Coomassie Brilliant Blue R250 to determine if the anti-CD22 mAbs were conjugated with DOX–platelet. Western blot was performed according to standard protocols to identify the platelet membrane proteins CD41, CD47, and CD61 [35]. Potential changes in platelet morphology were analyzed by SEM. The sizes of platelets, DOX–platelet, and DOX–platelet–CD22 were determined by dynamic light scattering. A platelet aggregation assay with adenosine diphosphate induction was performed and evaluated by spectrophotometry to assess the activation of DOX–platelet–CD22 cells.

The *in vitro* release behavior of DOX from DOX–platelet–CD22 was estimated by dynamic dialysis. Briefly, 5 mL of DOX–platelet–CD22 was enclosed in dialysis bags, which were then submerged in PBS at pH 5.5, 7.4, and 8.4. The bags were incubated under constant shaking (100 rpm) at 37°C. Up to 2 mL of PBS was removed every 2 h. DOX concentration was quantified by fluorescence spectrophotometry.

Confocal microscopy was used to visually determine successful drug loading and CD22 conjugation on the platelet surface. Raji cells were treated with DOX, DOX–platelet, and DOX–platelet–CD22 for 24 h. Then, the cells were centrifuged, washed, and resuspended in PBS. One drop of the suspension was placed on a glass slide. The sample was observed under a laser scanning confocal microscope after DAPI staining.

### Fluorescence intensity of intracellular doxorubicin

Cellular uptake was quantitatively detected by FCM to determine if the intracellular concentration of DOX increased in DOX–platelet- and DOX–platelet–CD22-treated cells. Briefly, different cells (Raji, Mino, Jurkat, and PBMC) were incubated for 24 h with DOX–platelet or DOX–platelet–CD22. The cells were washed twice, and then analyzed via FCM. The autofluorescence of DOX is red and the relative fluorescence intensity was calculated as FI_treated cells_/FI_control cells_.

### Cell viability assay

IC_50_ values for DOX, DOX–platelet, and DOX–platelet–CD22 were previously determined as 0.242, 0.192, and 0.09 μg/mL (Supplementary Figure 3), respectively. Therefore, 0.242 μg/mL of DOX was used and the equivalent dose of DOX in different forms was used for the subsequent *in vitro* experiments. The CCK-8 assay was performed to measure cell viability. Briefly, different cells (Raji, Mino, Jurkat, and PBMC) were treated with PBS, native platelets, anti-CD22 mAbs, platelet–CD22, DOX, DOX–platelet, and DOX–platelet–CD22 in a 96-well plate and incubated for 24, 48, or 72 h. CCK-8 solution (10 μL) was then added to each well and incubated for 3 h. The optical density (OD) of the wells at 450 nm was read in a microplate reader. Cell viability (%) was calculated as OD_treatment_/OD_control_ × 100%. Cell inhibition rate (%) was calculated as (1 − OD_treatment_/OD_control_) × 100%.

### Analysis of apoptosis

Raji cells were exposed to DOX, DOX–platelet or DOX–platelet–CD22 for 24 h. The cells were then washed with PBS and resuspended in binding buffer. Apoptotic cells were stained by annexin V-FITC and *propidium iodide* (PI) according to the manufacturer's instructions, and then analyzed by FCM.

Raji cells that were subjected to the different treatments were also stained with DAPI. Any morphological changes in the nuclei were observed under a fluorescent microscope.

### Cell cycle analysis

The Cycletest− Plus DNA Reagent Kit from BD Biosciences was used in this experiment. Raji cells that were treated with different agents for 24 h were centrifuged at 1,000 rpm for 5 min and washed with PBS. The cells were stained with DNA Prep Reagents kit following the manufacturer's protocols. Briefly, 250 μL of solution A was added to the precipitate and incubated for 10 min at room temperature. Then, 200 μL of solution B was added and the precipitate was incubated for an additional 10 min. Subsequently, 200 μL of solution C was added and the precipitate was stored at 4°C for 10 min away from light. The fluorescence intensity of the final mixture was analyzed by FCM.

### Western blot analysis

Western blot was performed in accordance with standard protocols [35]. Briefly, total proteins of Raji cells that were subjected to different treatments were extracted on ice using RIPA buffer (150 mM NaCl, 50 mM Tris–HCl pH 8, 0.5% sodium deoxycholate, 1% NP-40, 0.1% *sodium dodecyl sulfate* (SDS)). Proteins (24 μg) were separated by SDS polyacrylamide gel electrophoresis, transferred to a polyvin nitrocellulose membrane, and blocked for 1 h with 5% *bovine serum albumin* (BSA). Subsequently, the protein bands were incubated with Bax, Bcl-2, cleaved caspase-3, cleaved caspase-9, and β-actin primary antibodies. Horseradish peroxidase (HRP)-conjugated IgG was used as the secondary antibody. The bands were detected by an enhanced chemiluminescence detection system.

### Distribution of DOX-platelet-CD22 *in vivo*

To investigate the effects of DOX–platelet–CD22 *in vivo*, we first established a tumor-bearing mouse model via subcutaneous injections with 2 × 10^7^ Raji cells/mL in 200 μL of PBS. When tumor sizes reached 80–150 mm^3^, the mice were randomly assigned into three groups: (1) DOX, (2) DOX–platelet, and (3) DOX–platelet–CD22. Each mouse received 200 μL of the treatment agents (8 mg/kg DOX for the detection of its autofluorescence) via intravenous administration. The mice were imaged with DOX autofluorescence (excitation wavelength of 485 nm and emission wavelength of 585 nm) and FITC-labeled anti-CD22 mAbs (excitation wavelength of 488 nm, emission wavelength of 525 nm). The mice were anesthetized and imaged using a Caliper IVIS (PerkinElmer) at 24 h after injection.

### Plasma concentration of DOX in tumor-bearing mice

Tumor-bearing mice were intravenously administered with free DOX, DOX–platelet or DOX–platelet–CD22 (DOX 5 mg/kg) with three mice in each group. Blood samples were extracted from the tail vein at different time points using an anticoagulation tube. The whole blood was centrifuged at 3000 rpm for 10 min to obtain plasma. Plasma DOX concentrations were determined by spectrofluorometry (excitation wavelength: 485 nm, emission wavelength 590 nm).

### Experimental therapy of DOX–platelet–CD22 *in vivo*

The tumor-bearing mice were randomized to seven groups (DOX 1.2 mg/kg) as follows: (1) PBS (control); (2) native platelets; (3) anti-CD22 mAbs; (4) platelet–CD22; (5) DOX; (6) DOX–platelet; and (7) DOX–platelet–CD22. The length (a) and width (b) of the tumors were measured every two days. The tumor volume (V) and relative tumor volume (RTV) were calculated as follows: V=ab^2^/2 and RTV=V/V1 (V1 represents the initial tumor volume before treatment). Plasma levels of CK-MB, ALT, and BUN were determined on the 12th day post-treatment. All mice were sacrificed and their tumors and organs were removed for further analysis.

### TUNEL staining

TUNEL staining was performed using a commercial kit (*in situ* Cell Death Detection Kit, Fluorescein; Roche) with the recommended protocol [36]. Frozen sections of tumor tissues were prepared and rinsed twice with PBS. Once the area around sample had dried, 50 μL TUNEL reaction mixture (containing TdT and dUTP) was added to the sample. The sample was then incubated in a humidified atmosphere for 60 min at 37°C in the dark. Sections were rinsed thrice with PBS, embedded with antifade, and then analyzed under a fluorescence microscope (excitation wavelength: 450–500 nm; emission wavelength: 515–565 nm).

### Determination of ROS levels

FCM analysis was performed to visualize the total intracellular levels of ROS in myocardial cells. Myocardial cells were isolated from mouse hearts with different treatments. Cells were filtered, centrifuged, washed, and immediately incubated with H2DCFDA (2.5 mM) for 30 min for FCM analysis.

### Histopathological analysis

Mouse hearts, livers, lungs, spleens, and kidneys were carefully removed, fixed in 4% paraformaldehyde solution, and embedded in paraffin blocks. Tissue sections were prepared and stained with hematoxylin–eosin for routine histopathological examination under an optical microscope.

### Immunohistochemical staining

The paraffin-embedded sections of spleens from each group were deparaffinized, retrieved, blocked, and then incubated with anti-CD19 mAbs overnight at 4°C in a humidified box, followed by incubation with biotinylated secondary antibodies for 30 min at room temperature. Afterwards, streptavidin-HRP was added with incubation for 30 min. After the addition of 3,3′-*diaminobenzidine* tetrahydrochloride, the slides were then viewed under a microscope to observe color change (2–10 min). Hematoxylin was used for counterstaining.

### Immunofluorescence assay

Tumors were carefully removed and prepared as frozen sections at an ultra-low temperature. Room-temperature sections were washed with PBS thrice. The sections were blocked with 5% BSA supplemented with 0.3% Triton-X 100 for 1 h at room temperature. Bax, Bcl-2, cleaved caspase-3, or cleaved caspase-9 primary antibodies were added to the sections. The sections were then incubated at 37°C for 1 h in a wet box after the residual liquid was removed. IgG–FITC, which was used as the secondary antibody, was added dropwise to the samples. The samples were then incubated for 1 h in the dark. The cell nuclei were stained by DAPI. The sections were covered with antifade mounting medium and observed under a fluorescent microscope.

### Statistical analysis

Data are presented as the mean ± standard deviation. Student's *t*-test was used to analyze the data (SPSS software, Version 13.0; Chicago, US). A *P* value less than 0.05 was considered statistically significant.

## SUPPLEMENTARY MATERIALS FIGURES


