# Supplementary material for: Doxorubicin-loaded platelets conjugated with anti-CD22 mAbs: a novel targeted delivery system for lymphoma treatment with cardiopulmonary avoidance
Source: Oncotarget. 2017 Apr 6;8(35):58322–37. doi: 10.18632/oncotarget.16871 (PMC5601655; doi:10.18632/oncotarget.16871)
Supplement: Supplementary file 1 [file oncotarget-08-58322-s001.pdf]

Doxorubicin-loaded platelets conjugated with anti-CD22 mAbs: a novel targeted delivery system for lymphoma treatment with cardiopulmonary avoidance

SUPPLEMENTARY FIGURES

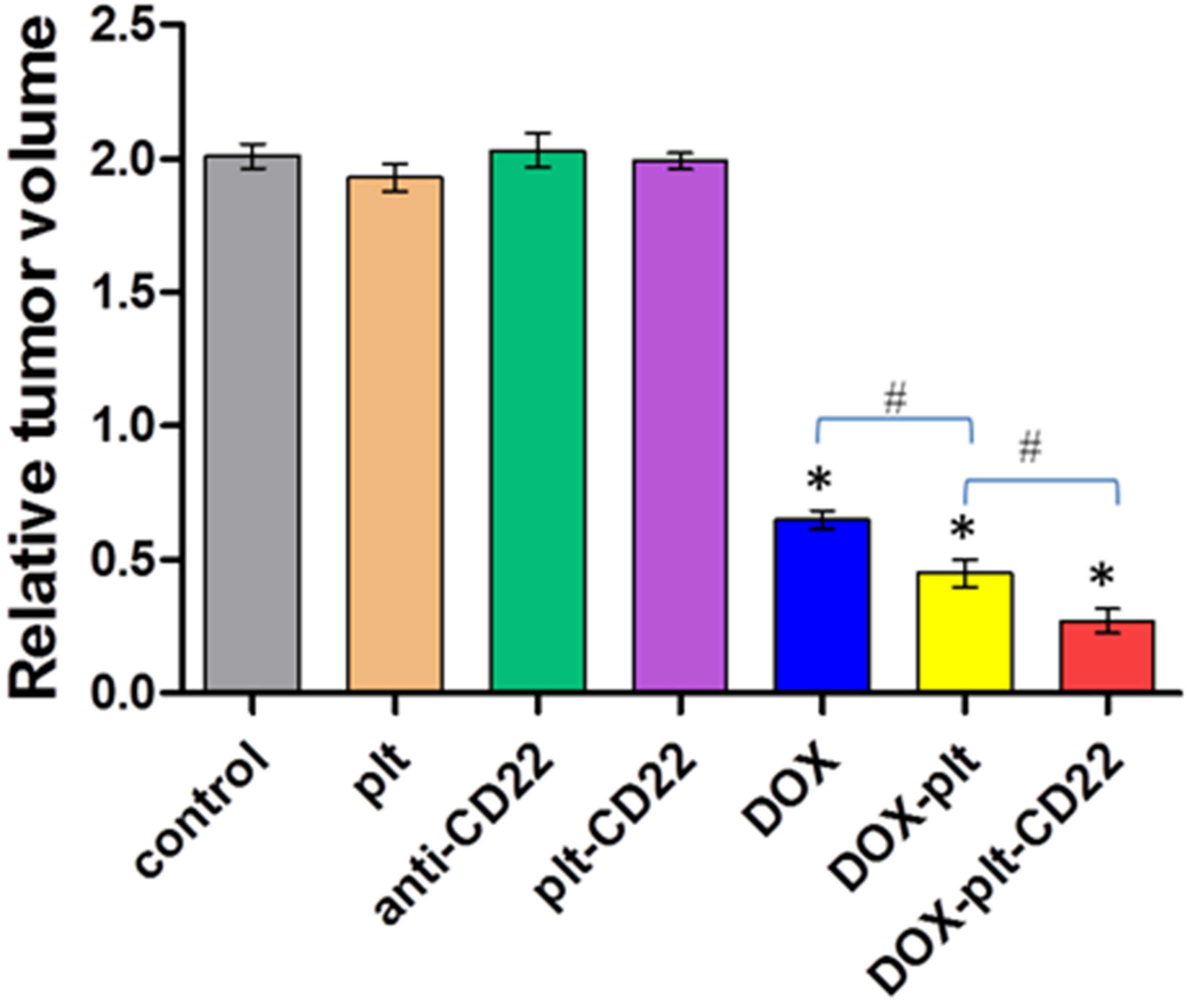

Supplementary Figure 1: The final relative volume of tumors from mice with different treatments. The final volumes of tumors from the DOX–platelet–CD22 group were smallest (*$P<0.05$ when compare with controls, #$P<0.05$).

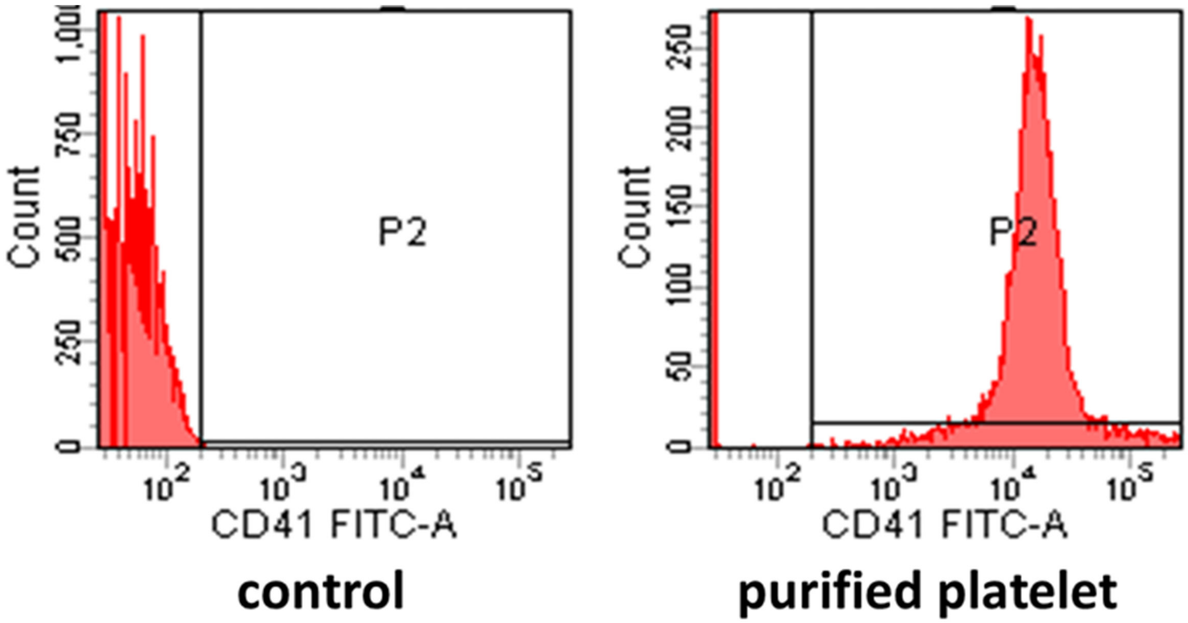

Supplementary Figure 2: Purity of platelet population detected by FCM analysis. The purity exceeds 96%.

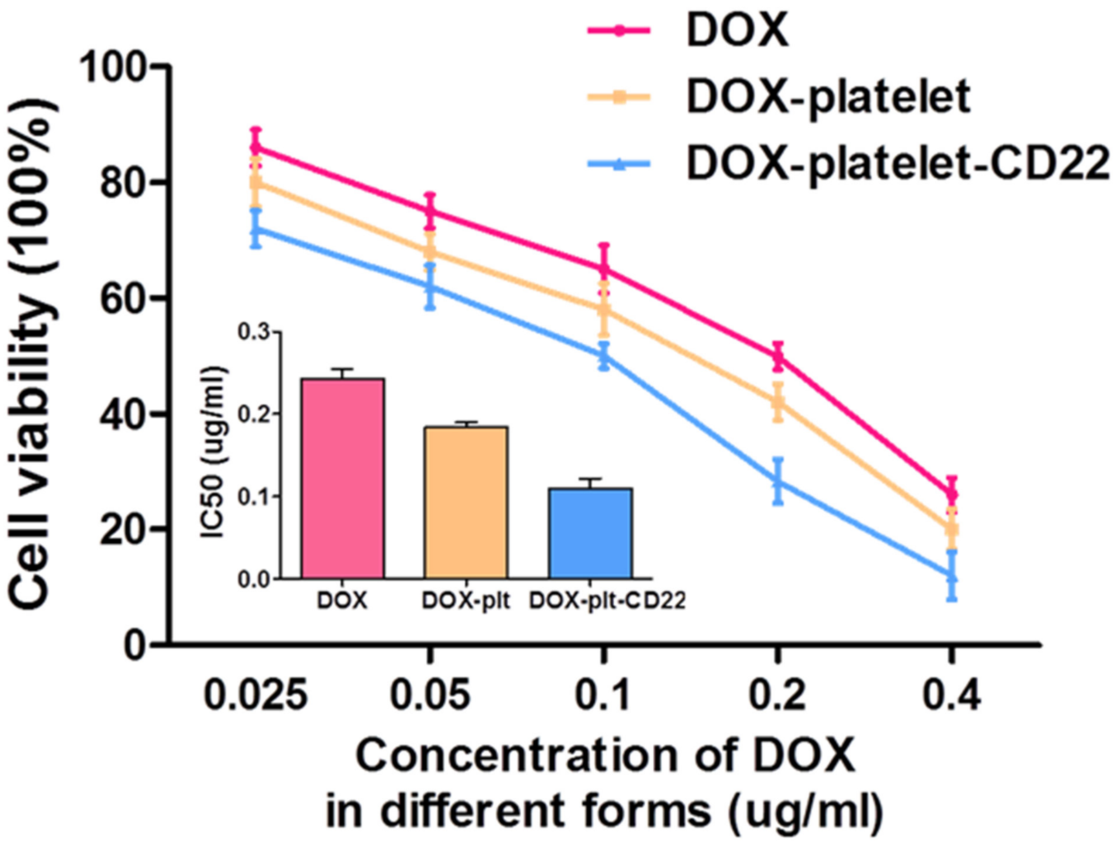

Supplementary Figure 3: The cytotoxic effect of DOX, DOX–platelet and DOX–platelet–CD22 against Raji cells with different concentration of DOX; Inset: The IC50 of DOX in different forms for Raji cells. The IC_{50} values for DOX, DOX–platelet, and DOX–platelet–CD22 are 0.242, 0.192, and 0.09 μg/mL, respectively.
